# INTERGROWTH 21st versus GROW fundal height charts and antenatal detection of small‐for‐gestational age: A multicenter cohort study

**DOI:** 10.1002/ijgo.71128

**Published:** 2026-06-09

**Authors:** Emily Butler, Hanna Ellson, Hannah Taylor, Oliver Hugh, Jason Gardosi

**Affiliations:** ^1^ Perinatal Institute Birmingham UK; ^2^ University of Warwick Coventry UK

**Keywords:** fetal growth, GROW, growth charts, INTERGROWTH, small‐for‐gestational age, symphysis‐fundal height

Serial assessment of fundal height is an important screening tool in low‐risk pregnancies, for the identification of fetuses requiring referral for assessment of fetal size and growth by ultrasound biometry and Doppler. It is an essential part of guidelines for fetal growth surveillance,^1^ and usefulness in detection of small‐for‐gestational age (SGA) fetuses has recently been confirmed in a randomized controlled trial.^2^ As a clinical tool, standardized fundal height (SFH) assessment requires training, and measurements need to be plotted on charts suitable for the local population and adjustable to maternal characteristics including parity and weight in early pregnancy.^3^, ^4^

The National Health Service (NHS) in England recently issued a directive^2^ against use of the INTERGROWTH 21st fetal weight charts because of their low antenatal screen positive rate for SGA.^5^ The widely promoted INTERGROWTH‐21 (IG21) standard for symphysis‐fundal height (SFH)^6^ is based on the same eight‐country cohort as their fetal weight standard (Brazil, China, India, Italy, Kenya, Oman, UK and US), and is also claimed to be valid universally. We investigated how this SFH standard performs when applied to our population.

The study cohort consisted of 90 601 consecutive low risk pregnancies in 43 NHS hospitals between 2023 and 2025 that had three or more third trimester SFH measurements. All data were recorded with mothers' informed consent and then fully anonymized. Research with such cohort data was approved by the NHS Health Research Authority (IRAS Project ID 365709, REC 25/EM/0271). The IG21 SFH standard^6^ and the GROW SFH standard^7^ were used to compare SGA (<10th centile) antenatal screen positive rates and to calculate sensitivity (detection rate) of neonatal SGA. Proportions and means were compared and *P* values calculated using two‐proportions Z test and two‐sample *t*‐tests, respectively.

Table 1 compares the pregnancy characteristics in our NHS cohort with the cohort on which the IG21 standard is based.^6^, ^8^ There were significant differences in nine of the 10 categories in which data was available for both cohorts, including mothers' ethnicity, height, body mass index (BMI, calculated as weight in kilograms divided by the square of height in meters) and weight of their babies at birth. The NHS cohort had an average of 4.6 third‐trimester fundal height measurements per pregnancy, with a median of 33^+6^ weeks (interquartile range [IQR]: 29^+3^–36^+3^). The rate of SGA by GROW and IG21 was 8.7% and 5.0%, respectively, which represents a 42% lower screen positive rate according to IG21.

**TABLE 1:** Comparison of a UK study cohort and eight‐country Intergrowth^6^ cohort.

	Current cohort	Intergrowth	*P* value
Pregnancies	90 601	4239	
Maternal age (years), mean (SD)	29.9 (4.9)	28.4 (3.9)	<0.01
Maternal height (cm), mean (SD)	164.2 (6.7)	162.2 (5.8)	<0.01
Maternal weight (kg), mean (SD)	68.2 (12.0)	61.3 (9.1)	<0.01
BMI, mean (SD)	25.3 (4.0)	23.3 (3.0)	<0.01
Ethnicity British, *n* (%)	57 572 (63.5)	606 (14.3)^a^	<0.01
Nulliparous, *n* (%)	50 855 (56.1)	2907 (68.6)	<0.01
Male infant, *n* (%)	46 068 (50.9)	2106 (49.7)	0.14
Term LBW <2500 g (≥37 weeks), *n* (%)	1106 (1.2)	127 (3.0)	<0.01
Preterm birth (<37^+0^ weeks), *n* (%)	1891 (2.1)	192 (4.5)	<0.01
Birth weight ≥37 weeks (g), mean (SD)	3468 (440)	3267 (444)	<0.01

*Note*: BMI, calculated as weight in kilograms divided by the square of height in meters.

Abbreviations: BMI, body mass index; LBW, low birth weight; SD, standard deviation.

^a^ Based on ethnicity distribution in Villar et al.^8^

In total, 6727 (7.4%) babies in this low‐risk population receiving serial fundal height measurements were born SGA, and this would have been flagged antenatally by one or more SFH measurements in 1732 (25.7%) pregnancies according to both GROW and IG21 SFH standards. An additional 84 (1.2%) of pregnancies with an SGA neonate would have been identified by the IG21 SFH standard and not by GROW, resulting in an overall IG21 detection rate of 27.0%. A further 822 (12.2%) pregnancies were SGA by GROW only, resulting in an overall detection rate of 38.0% by GROW and indicating that IG21 would have missed 822 of 2554 (32.2%) of SGA neonates.

In conclusion, the INTERGROWTH SFH chart^6^ is derived from pregnancies of mothers with lower average parity, early‐pregnancy weight and term birth weight than those in the NHS population (Table 1). This results in a lower normal range and SGA cutoff for fundal height, and is the probable reason for the 42% lower screen‐positive rate with IG21. As a consequence, antenatal identification of SGA birth weight and risk of fetal growth restriction is likely to be missed in about one third of pregnancies monitored with IG21 SFH charts in the NHS. We recommend that, as with EFW, the appropriateness and safety of a chart should be tested before use in the local population.

## AUTHOR CONTRIBUTIONS

Conceptualization: JG, OH. Data analysis and verification: OH. Initial draft: JG. All authors had access to the data and reviewed, edited and approved the final draft.

## CONFLICT OF INTEREST STATEMENT

All authors are employees of the Perinatal Institute, a not‐for‐profit social enterprise that has developed and licenses the GROW fetal growth chart referred to in this article.

## Data Availability

The data that support the findings of this study are available from the corresponding author upon reasonable request.
